# Response of phytoplankton composition to environmental stressors under humidification in three alpine lakes on the Qinghai-Tibet Plateau, China

**DOI:** 10.3389/fmicb.2024.1370334

**Published:** 2024-04-15

**Authors:** Peiwen Gu, Junmei Jia, Delin Qi, Qiang Gao, Cunfang Zhang, Xi Yang, Miaomiao Nie, Dan Liu, Yule Luo

**Affiliations:** ^1^State Key Laboratory of Plateau Ecology and Agriculture, Qinghai University, Xining, China; ^2^College of Eco-Environmental Engineering, Qinghai University, Xining, China

**Keywords:** phytoplankton, succession, biodiversity, environmental response, eutrophication, alpine lakes

## Abstract

Owning to their extreme environmental conditions, lakes on the Qinghai-Tibet Plateau have typically displayed a simplistic food web structure, rendering them more vulnerable to climate change compared to lakes in plains. Phytoplankton, undergoing a changing aquatic environment, play a crucial role in the material cycle and energy flow of the food chain, particularly important for the unique fish species of the Tibetan Plateau. To identify the changing environment indexes and determine the response of phytoplankton composition to the environment change in alpine lakes, three lakes—Lake Qinghai, Lake Keluke and Lake Tuosu—were selected as study areas. Seasonal sampling surveys were conducted in spring and summer annually from 2018 to 2020. Our findings revealed there were significant changes in physicochemical parameters and phytoplankton in the three lakes. Bacillariophyta was the predominant phytoplankton in Lake Qinghai from 2018 to 2020, with the genera *Synedra* sp., *Navicula* sp., *Cymbella* sp. and *Achnanthidium* sp. predominated alternately. Lake Keluke alternated between being dominated by Bacillariophyta and cyanobacteria during the same period. *Dolichospermum* sp., a cyanobacteria, was prevalent in the summer of 2018 and 2019 and in the spring of 2020. In Lake Tuosu, Bacillariophyta was the predominant phytoplankton from 2018 to 2020, except in the summer of 2019, which was dominated by cyanobacteria. *Synedra* sp., *Oscillatoria* sp., *Pseudoanabaena* sp., *Chromulina* sp. and *Achnanthidium* sp. appeared successively as the dominant genera. Analysis revealed that all three lakes exhibited higher phytoplankton abundance in 2018 that in 2019 and 2020. Concurrently, they experienced higher average temperatures in 2018 than in the subsequent years. The cyanobacteria, Bacillariophyta, Chlorophyta and overall phytoplankton increased with temperature and decreased with salinity and NH_4_-N. Besides, the ratios of cyanobacteria, and the ratios of Bacillariophyta accounted in total phytoplankton increased with temperature. These findings suggest that cyanobacteria and phytoplankton abundance, especially Bacillariophyta, may have an increase tendency in the three alpine lakes under warm and wet climate.

## 1 Introduction

Phytoplankton serve as primary producers in aquatic ecosystems, playing a critical role in the material cycle and energy flow of food chains (Zhang et al., 2021). Biological production serves as an indicator of the trophic status and potential of fishery resources in aquatic organisms. The productivity of an aquatic environment is closely linked to the density of phytoplankton (Jakhar, 2013). The structure of the phytoplankton community is a sensitive indicator for assessing water quality changes, and its composition offers valuable insights into these shifts (Ger et al., 2014). A large amount of nutrients is discharged into freshwater systems along with surface runoff and domestic sewage (Carpenter et al., 1998a). Consequently, the phytoplankton community becomes overpopulated, aquatic plants gradually diminish or even disappear, and cyanobacterial blooms drive the deterioration of water quality and aquatic ecosystems in eutrophic lakes worldwide (Conley et al., 2009). The degradation of water resources through eutrophication can lead to the loss of the amenities or services that these aquatic resources provide (Smith, 2003). Climate change accelerated the degradation rate in aquatic systems (Huang et al., 2021). Qinghai-Tibetan Plateau is one of the most sensitive regions to global warming worldwide. Even though the rare species on the Tibetan Plateau has received widespread attention due to its unique geographical location and climatic conditions, the primary producers phytoplankton was seldom considered in those areas.

Extensive researches had done to explore driving factors of phytoplankton succession under climate change (Winder and Sommer, 2012; Li et al., 2016; Cao et al., 2018; Li et al., 2023). Temperature was one of the most important drivers. High temperatures favor phytoplankton growth, since temperature affects life activities such as metabolism by influencing enzyme activity in phytoplankton. Phytoplankton had different temperature optimum. Bacillariophyta are able to grow rapidly when temperatures are low, while cyanobacteria and Chlorophyta may become dominant genera in summer as temperatures rise (Chen et al., 2003; Ke et al., 2008). Beyond temperature, nutrients are indispensable for phytoplankton growth and reproduction, and appropriate concentrations will promote phytoplankton growth and reproduction. Commonly, nutrient enrichment fosters the growth of specific phytoplankton like *Microcystis*, *Dolichospermum* sp., *Nostoc* and *Aphanizomenon*, which can be detrimental to both aquatic and terrestrial life (Chen et al., 2008; Lv et al., 2011). Nitrogen (N) and Phosphorus (P) are often identified as the primary nutrients limiting aquatic algal growth (Lv et al., 2011). The P essential for summer algal blooms originates from rapid water column P cycling and sediment P release, a process accelerated by increased salinity, which supplies sulfate reducing sediment *microorganism* populations and triggers substantial P release (Conley, 1999; Blomqvist et al., 2004). Long-term analysis in Lake Geneva demonstrated that phytoplankton photosynthetic efficiency is directly proportional to water temperature given adequate phosphorus, a relationship amplified at elevated nutrient concentrations. In phosphorus-limited scenarios, the correlation between photosynthetic efficiency and temperature diminishes (Tadonléke, 2010). Excessive P in the water column causes cyanobacterial blooms in the summer. Concurrently, N is essential for the growth of cyanobacteria. Phytoplankton communities experience regular variation in response to nutrient stress. Previous studies have shown that nitrogen deficiency induces nitrogen fixation by phytoplankton, favoring the growth and reproduction of nitrogen-fixing algae. This in turn favors the growth and reproduction of nitrogen-fixing algae (Dokulil and Teubner, 2000). In a high mountain lake, nutrient enrichment bioassays indicated that phytoplankton growth was severely limited by phosphorus due to overgrazing by pastoralists and ongoing climate change (Ren et al., 2019; Jia et al., 2022). In contrast, nitrogen did not significantly impact phytoplankton growth, but excessive nitrogen is toxic to phytoplankton. In addition, phytoplankton growth is affected by environmental factors such as salinity and NH_4_-N and is able to react quickly by responding to changes in the environment, which some previous studies might have overlooked. Each species of planktonic plants has a specific salinity range optimal for its growth and reproduction. Therefore, the variations of physicochemical parameters such as temperature, salinity and nutrient may favor some of phytoplankton genera and become stressors for others under climate change.

The Qinghai-Tibet Plateau, known as the world’s highest and most expansive plateau, encompasses a significant cluster of plateau lake systems (Li et al., 2021). The Tibetan Plateau is very sensitive to climate change and the lakes undergone expansion on surface water in recent years (Lei and Yang, 2017). This situation may alters the physico-chemical properties of water bodies and lake nutrient concentrations since increasing terrestrial inflow (Liu, 2013; Lu et al., 2017). These changes in environmental factors will inevitably have a profound impact on the composition and structure of phytoplankton (Abirhire et al., 2015; Jia et al., 2022). Previous studies had demonstrated that cyanobacteria were boosted in many aquatic systems under climate change. Alpine lakes, characterized by their special geographical location and extreme environmental conditions, form an integral part of mountain systems and typically feature simpler aquatic ecosystems compared to other lakes. Lakes like Lake Qinghai, Lake Keluke and Lake Tuosu, all situated on the Tibetan Plateau, are distinguished by their extreme conditions and presented low phytoplankton abundance without blooms. Although alpine lakes have a low percentage of cyanobacteria, cyanobacteria may increase as the increase of temperatures and nutrients load and pose some threat to rare fish and birds in the lakes under climate change. In recent years, Lake Qinghai, Lake Keluke and Lake Tuosu all had undone an marked increase of surface water. For example, the water level of Lake Qinghai decreased by 3.35 m, 2.77 m and 0.58 m from 1959 to 2000, 1959 to 1986, and 1986 to 2000, respectively. Compared to 1989, the water area of Lake Qinghai had shrunk by 129 km^2^ in 2004. The lake’s area has expanded due to climate-induced increases in rainfall since 2005, reaching 4,476 km^2^ in 2017, and it is expected that the water area will continue to increase from 2021 to 2050 in Lake Qinghai (Li et al., 2007; Dong et al., 2018). This had resulted in a series of ecological environmental problems in littoral zone of Lake Qinghai, for example the blooming of Cladophora (Wu et al., 2023). However, seldom had research focused on the changes in aquatic environment and the phytoplankton on the Tibetan Plateau. In present study, we intended to identify those changing aquatic indexes and phytoplankton succession in the typical alpine lakes under climate change, and response of phytoplankton communities to changing aquatic indexes. This research would provide the basic information of sensitive environment indexes to climate change in alpine lakes and the response of phytoplankton composition to the environment change, which is imperative for the lake protection on the Tibetan Plateau under climate change.

## 2 Materials and methods

### 2.1 Study area and sampling locations

**Figure 1:**
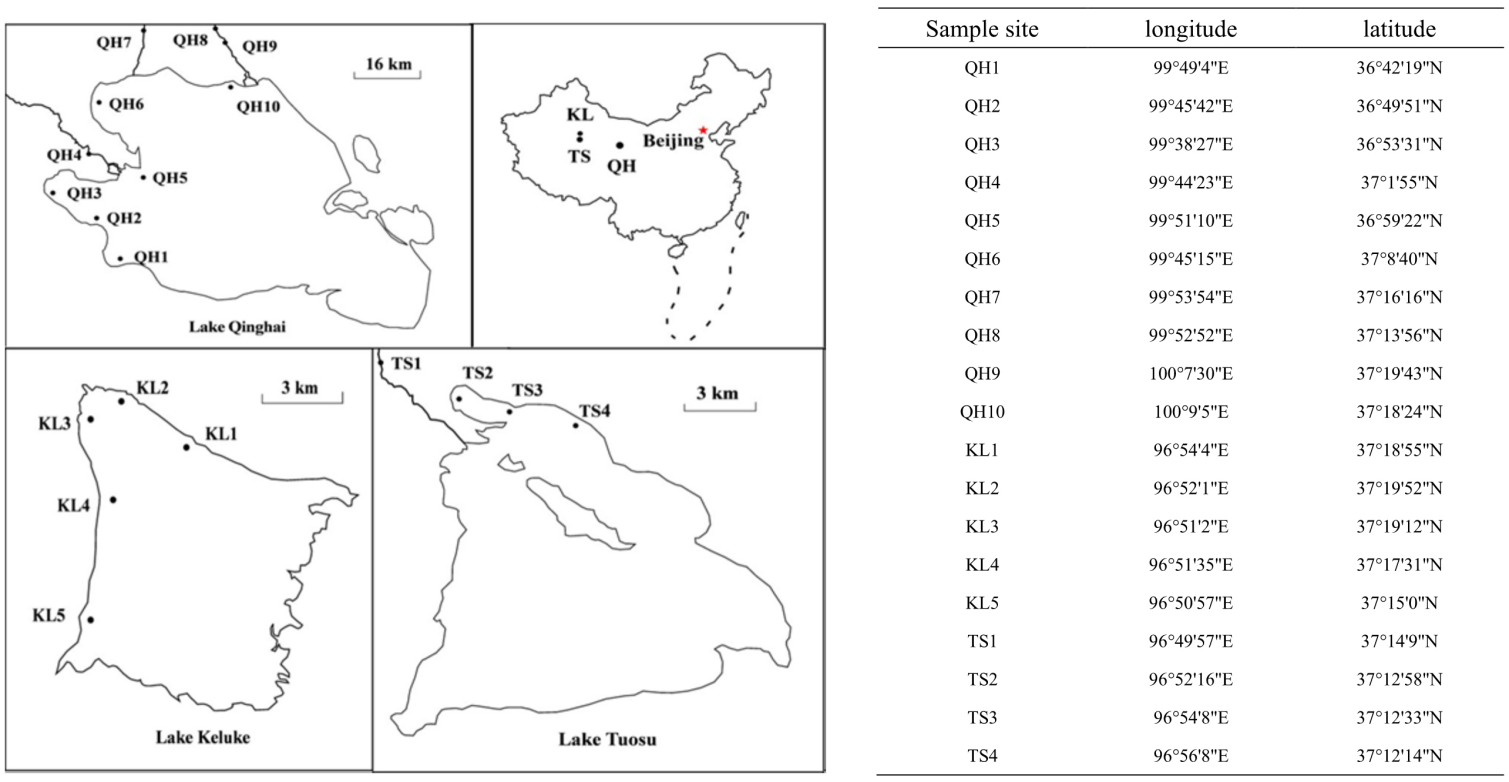
Sampling sites of Lake Qinghai, Lake Keluke, and Lake Tuosu, in China.

Lake Qinghai (36°32′–37°15′N; 99°36′–100°16′E) is located in northeastern (Jia et al., 2022) Qinghai-Tibetan Plateau, and is the largest lake in China (Figure 1). It covers an area of approximately 4,300 km^2^ (Tang et al., 2018), has a maximum depth of 30 m, and a mean surface elevation of 3,194 m. The lake is bordered by Qilian Mountains, AltynTagh Mountains, and the Kunlun Mountains (Abirhire et al., 2015; Li et al., 2021). This region has an alpine and continental climate with an annual average temperature of 1.2°C (Cao et al., 2020). With the unique beauty of the plateau lakes, it is known as the “China’s most beautiful lake” and is a famous tourist destination in China. With the increase in popularity of Qinghai Lake tourism, the number of tourists has been increased year by year, greatly contributing to Qinghai Province’s economic growth. However, tourism has brought economic benefits, but also the ecological environment of the tourist sites have been damaged by the increasing tourists (Zhang, 2019). Lake Keluke (37°10′–37°20′N; 96°49′–97°30′E) is a small salty water in Qinghai Province and the habitat and breeding place of many important waterfowls (Tang and Ju, 2011). It spans an area of about 56.7 km^2^, boasts a mean water depth of 4 m, and reaches a maximum depth of 13.3 m. The lake is rich in Chinese mitten crabs, or hairy crabs, and is also dominated by the habitat of crane birds. Due to the large number of people living in the neighborhood of Lake Qinghai and Lake Keluke, the area of farming in the reserve is also large. The density of settlements directly affects the habitat of birds and destroys the aquatic vegetation of wetlands, while surface farming has a tendency to cause eutrophication of lake water. Around the lake area, tens of thousands of livestock graze annually, and a large amount of animal manure is washed into the lake, making the lake mud and water very fertile, providing nutritious growth conditions for aquatic plants, and also causing a certain degree of water pollution (Guo, 2013). Lake Tuosu (37°04′–37°13′N; 96°50′–97°03′E) is situated in the east of the Qaidam Basin, holding an average elevation of 2,800 meters. Its annual average temperature at 3°C (Song and Sun, 2020). Lake Tuosu primarily receives water from Lake Leluke, with the two lakes being linked by a river (Chen et al., 2003). Lake Keluke and Lake Tuosu have the same ecological environment and history of change but had different characteristic. Lake Tuosu is a typical inland saltwater lake and surrounded by the vast Gobi Desert. The temperature is relative high and the water evaporation of the lake is very high. There is very little aquatic flora and fauna in Lake Tuosu (Sheng, 2022).

### 2.2 Sample collection and processing

Water samples of Lake Qinghai, Lake Keluke and Lake Tuosu were collected during the spring and summer of 2018 (Jia et al., 2022), 2019 and 2020. Samples were taken from 10, 5 and 4 sites in Lake Qinghai, Lake Keluke and Lake Tuosu, respectively (Figure 1). The sampling time is listed in Table 1. Surface water samples, from a depth of 0.5 meters, were collected in 1 L plastic containers. All samples were stored in polyethylene barrels at 4°C, kept cool and shaded, and transported to the laboratory for analysis within 24 h (Ke et al., 2008). Analysis of the water parameters for the three lakes included temperature, pH, salinity, TN, TP and NH_4_-N. Water temperature, pH and salinity were measured on-site at each location using a YSI 6600 multi-probe sonde (YSI, Yellow Springs, OH, United States). Total nitrogen (TN) and total phosphorus (TP) were measured using Chinese standard methods HJ/T 636-2012 and GB/T 11983-1989, respectively. NH_4_-N was measured following the Chinese standard method HJ 535-2009.

**Table 1:** The sample collection times in the three lakes in China.

Years	Season	Lake Qinghai	Lake Keluke	Lake Tuosu
2018	Spring	28-29^th^ May	1^th^ June	2^th^ June
	Summer	20-21^th^ August	18^th^ August	19^th^ August
2019	Spring	10-11^th^ June	8^th^ June	9^th^ June
	Summer	1-2^th^ September	30^th^ August	31^th^ August
2020	Spring	10-11^th^ June	8^th^ June	9^th^ June
	Summer	10-11^th^ September	8^th^ September	9^th^ September

Quantitative water samples were stored in clean plastic containers, and phytoplankton genera were fixed with Lugol’s iodine solution (1.5% v/v). Phytoplankton were allowed to settle for 48 h. Based on monographs, phytoplankton were identified and enumerated under 20 × 20 magnification using light microscopy for cell density (Lv et al., 2011; Zhang et al., 2016). The Shannon-wiener index (H) of phytoplankton diversity were computed (Chen et al., 2008). Biodiversity of phytoplankton in the three lakes was calculated according to groups such as cyanobacteria, Bacillariophyta, Chlorophyta, Cryptophyta, Euglenophyta, Pyrrophyta and Chrysophyta (Liu et al., 2016; Li et al., 2021).

### 2.3 Statistical analysis

All physical and chemical parameters were analyzed using SPSS software, version 26.0 for Windows (Chicago, United States). The data approximately conformed to a normal distribution. Analysis of variance was employed to assess the differences in water parameters and phytoplankton both temporally and spatially. RDA analyzed was applied to assess relationships between phytoplankton and environmental factors using canoco 5. Detrended correspondence analysis (DCA) was conducted to assess the length of the dominant gradient of environmental factors and phytoplankton and the first axis of lengths of gradient was <3.0. Thusly, RDA analysis was chosen in present study. Linear regression models were applied to environmental indexes and phytoplankton based on yearly average data. Water parameters and phytoplankton showing a *p* value <0.05 were considered to be significantly different or related.

## 3 Results

### 3.1 Water parameter and nutrients in the three lakes

Table 2 displays the annual changes in water parameters and nutrient levels. Lake Qinghai had significantly higher temperature in 2018 than in 2020 (*p* < 0.05). Lake Keluke and Lake Tuosu recorded significantly higher temperatures in 2018 than in 2019 and 2020 (*p* < 0.05). Over the three-year period, Lake Tuosu had the highest average temperature, followed by Lake Keluke, with Lake Qinghai having the lowest (*p* < 0.05). In terms of salinity, Lake Tuosu exhibited the highest levels among the three lakes (*p* < 0.05), followed by Lake Keluke, with Lake Qinghai recording the lowest salinity (*p* < 0.05). There were notable differences in the NH_4_-N levels among the lakes. Lake Tuosu had the highest NH_4_-N, followed by Lake Keluke, while Lake Qinghai had the lowest. Compared to Lake Qinghai, Lake Tuosu demonstrated higher levels of temperature, salinity and NH_4_-N. The TP concentrations in all three lakes remained below 0.1 mg/L. Lake Keluke exhibited elevated TP levels compared to Lake Qinghai and Lake Tuosu. Fluctuations in TP concentrations between Lake Qinghai and Lake Tuosu over different years were minimal. Conversely, Lake Tuosu had higher TP concentrations than both Lake Qinghai and Lake Keluke during those years.

**Table 2:** Means and standard deviations of several water parameters of lakes in China.

Lakes	Years	Water parameters							
		Temperature (°C)	DO (mg/L)	C(mS/cm)	Salinity (ppt)	pH	TN (mg/L)	TP (mg/L)	NH_4_-N (mg/L)
Qinghai(12)	2018	15.0 ± 4.4	3.6 ±3.7	5.1 ±5.8	4.3 ±4.9	8.8 ±0.4	4.6 ±6.0	0.02 ±0.03	NA ± NA
	2019	13.4 ±5.3	5.8 ±1.8	5.9 ±6.9	4.2 ±4.9	8.2 ±2.5	3.8 ±3.4	0.06 ±0.09	1.5 ±2.5
	2020	10.4 ±6.9	4.7 ±3.1	4.9 ±6.8	3.5 ±5.0	6.8 ±4.4	0.9 ±0.8	0.03 ±0.04	0.2 ± 0.2
	Mean	13.0 ±5.9	4.7 ±3.1	5.3 ±6.5	4.0 ±4.9	7.9 ±3.1	3.1 ±4.2	0.04 ±0.06	0.6 ± 1.6
Keluke(6)	2018	20.8 ±2.5	3.6 ±4.0	6.0 ±13.1	5.6 ±13.2	8.4 ±0.2	4.6 ±4.9	0.04 ±0.04	NA ± NA
	2019	16.0 ±1.3	6.3 ±2.9	5.9 ±16.0	4.7 ±13.4	8.5 ±0.3	5.4 ±8.9	0.09 ±0.11	2.9 ±6.6
	2020	11.7 ±7.1	4.5 ±2.8	5.8 ±15.6	4.8 ±13.7	6.7 ±4.1	6.1 ±9.8	0.05 ±0.08	3.1 ±6.8
	Mean	16.2 ±5.7	4.8 ±3.4	5.9 ±14.5	5.0 ±13.0	7.9 ±2.4	5.4 ±7.9	0.06 ±0.0 8	2.0 ±5.5
Tuosu(4)	2018	21.1 ±2.5	3.7 ±4.0	12.9 ±10.5	11.7 ±10.1	8.9 ±0.3	1.4 ±1.0	0.02 ±0.006	NA ± NA
	2019	17.2 ±1.3	6.2 ±0.05	19.5 ±11.1	14.1 ±8.2	9.0 ±0.2	10.6 ±7.6	0.05 ±0.06	8.8 ± 6.9
	2020	18.8 ±3.0	6.1 ±0.3	19.3 ±11.3	13.3 ±7.8	9.5 ±0.2	6.8 ±4.7	0.03 ±0.04	4. 9±4.8
	Mean	19.0 ±2.8	5.4 ±2.5	17.2 ±11.0	13.0 ±8.4	9.1 ±0.3	6.3 ±6.3	0.03 ±0.04	4.6 ±5.9

### 3.2 Phytoplankton composition and biodiversity in the three lakes

**Figure 2:**
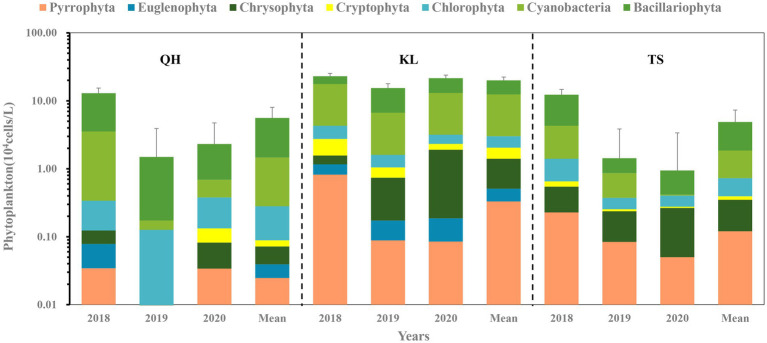
Phytoplankton composition based on the abundance in different seasons in Lake Qinghai (QH), Lake Keluke (KL), Lake Tuosu (TS). The standard deviations of phytoplankton abundance in each lake are indicated by the error bars.

The phytoplankton composition varied among the three lakes (Figure 2). Cyanobacteria, Bacillaiophyta, Chlorophyta, Pyrrophyta, Cryptophyta, Chrysophyta and Euglenophyta have been found both in Lake Qinghai and Lake Keluke. Cyanobacteria, Bacillaiophyta, Chlorophyta, Pyrrophyta, Cryptophyta and Chrysophyta were found in Lake Tuosu. Cyanobacteria, Bacillariophyta and Chlorophyta were dominated across all sampling sites in Lake Qinghai, Lake Keluke and Lake Tuosu in 2018, 2019 and 2020. Lake Qinghai recorded phytoplankton abundances of 1.3 × 10^5^ cells/L, 1.6 × 10^4^ cells/L and 2.9 × 10^5^ cells/L in 2018, 2019 and 2020, respectively. Within this lake, Bacillariophyta contributed to 73, 88 and 66% of the total, cyanobacteria made up 24, 3% and 14%. For Lake Keluke, the figures stood at 2.7 × 10^5^ cells/L, 1.8 × 10^5^ cells/L and 2.3 × 10^5^ cells/L for those respective years. Between 2018 and 2020, cyanobacteria represented 58%, 32%, and 46%, while Bacillariophyta made up 23%, 56%, and 46% of the phytoplankton. In Lake Tuosu, the numbers were 1.22 × 10^5^ cells/L, 1.43 × 10^4^ cells/L and 1.13 × 10^5^ cells/L over the 3 years. Bacillariophyta made up 65, 40 and 43% of the phytoplankton, cyanobacteria contributed 24%, 34%, and 2% in 2018, 2019, and 2020, and Chrysophyta accounted for 29% in 2020. Both Bacillariophyta and total phytoplankton abundance in Lake Qinghai were significantly higher in 2018 than in 2019 and 2020 (*p* < 0.05) and cyanobacteria in 2018 had a significantly higher abundance than in 2019 (*p* < 0.05). Lake Tuosu exhibited significantly higher Bacillariophyta abundance in 2018 compared to 2019 and 2020 (*p* < 0.05) along with a higher cyanobacteria abundance in 2018 than 2020 (*p* < 0.05).

Table 3 displays the percentage of dominant phytoplankton and genera. Bacillariophyta was dominanted in Lake Qinghai from 2018 to 2020. The *Synedra* sp., *Navicula* sp., *Cymbella* sp., *Synedra* sp., *Synedra* sp., and *Achnanthidium* sp. were emerging successively as the dominant genera, which accounted for 33%, 35%, 24%, 21%, 23%, and 41% of the total phytoplankton, respectively. In addition, there was a higher percentage of *Dolichospermum* sp. in spring 2018 and *Spirogyra* sp. in summer 2019 in Lake Qinghai, which accounted for 25% and 15% of the total phytoplankton. Cyanobacteria and Bacillariophyta were alternated dominanted in Lake Keluke during the same period, the genus *Dolichospermum* sp., was dominanted in the summer of 2018 and 2019 and in the spring of 2020, which accounted for 44%, 31%, and 32%, respectively. In Lake Tuosu, Bacillariophyta was dominant from 2018 to 2020, except in the summer of 2019, which was dominated by cyanobacteria, the dominant genera *Synedra* sp., *Oscillatoria* sp., *Pseudoanabaena* sp. and *Achnanthidium* sp. were appeared successively, which accounted for 28%, 26%, 23%, 24%, and 62%, respectively. Chlorophyta was the second dominant in the spring of 2020, which was dominated by the genus of *Chromulina* sp. accounted for 22% in Tuosu.

**Table 3:** Percentage of dominant phytoplankton genera in several lakes in China.

Years	Season	Lake Qinghai			Lake Keluke			Lake Tuosu		
		Dominant genera	Pi (%)	phytoplankton	Dominant genera	Pi (%)	phytoplankton	Dominant genera	Pi (%)	phytoplankton
2018	Spring	*Synedra* sp.	33	Bacillariophyta	*Synedra* sp.	21	Bacillariophyta	*Synedra* sp.	28	Bacillariophyta
		*Dolichospermum* sp.	25	Cyanobacteria	*Oscillatoria* sp.	21	Cyanobacteria	*Nitzschia* sp.	18	Bacillariophyta
		*Navicula* sp.	18	Bacillariophyta	*Navicula* sp.	11	Bacillariophyta	*Merismopedia* sp.	15	Cyanobacteria
	Summer	*Navicula* sp.	35	Bacillariophyta	*Dolichospermum* sp.	44	Cyanobacteria	*Synedra* sp.	26	Bacillariophyta
		*Synedra* sp.	17	Bacillariophyta	*Merismopedia* sp.	7	Cyanobacteria	*Dactylococcopsis* sp.	16	Cyanobacteria
		*Oscillatoria* sp.	8	Cyanobacteria	*Synedra* sp.	4	Bacillariophyta	*Chlorella* sp.	6	Chlorophyta
2019	Spring	*Cymbella* sp.	24	Bacillariophyta	*Synedra* sp.	34	Bacillariophyta	*Oscillatoria* sp.	23	Cyanobacteria
		*Synedra* sp.	18	Bacillariophyta	*Navicula* sp.	16	Bacillariophyta	*Synedra* sp.	15	Bacillariophyta
		*Navicula* sp.	10	Bacillariophyta	*Diatoma* sp.	16	Bacillariophyta	*Cymbella* sp.	15	Bacillariophyta
	Summer	*Synedra* sp.	21	Bacillariophyta	*Dolichospermum* sp.	31	Cyanobacteria	*Pseudoanabaena* sp.	24	Cyanobacteria
		*Spirogyra* sp.	15	Chlorophyta	*Oscillatoria* sp.	19	Cyanobacteria	*Dactylococcopsis* sp.	20	Cyanobacteria
		*Navicula* sp.	9	Bacillariophyta	*Pseudoanabaena* sp.	15	Cyanobacteria	*Synedra* sp.	19	Bacillariophyta
2020	Spring	*Synedra* sp.	23	Bacillariophyta	*Dolichospermum* sp.	32	Cyanobacteria	*Chromulina* sp.	22	Chlorophyta
		*Cyclotella* sp.	16	Bacillariophyta	*Synedra* sp.	28	Bacillariophyta	*Synedra* sp.	19	Bacillariophyta
		*Merismopedia* sp.	9	Cyanobacteria	*Oscillatoria* sp.	11	Cyanobacteria	*Cyclotella* sp.	7	Bacillariophyta
	Summer	*Achnanthidium* sp.	41	Bacillariophyta	*Fragilaria* sp.	35	Bacillariophyta	*Achnanthidium* sp.	62	Bacillariophyta
		*Fragilaria* sp.	20	Bacillariophyta	*Cyclotella* sp.	22	Bacillariophyta	*Fragilaria* sp.	17	Bacillariophyta
		*Cyclotella* sp.	1	Bacillariophyta	*Navicula* sp.	13	Bacillariophyta	*Gomphonema* sp.	9	Bacillariophyta

The phytoplankton biodiversity of the three lakes exhibited significant variances. In 2018, 2019 and 2020, Lake Qinghai’s phytoplankton diversity was 0.26 ± 0.33, 0.19 ± 0.31 and 0.19 ± 0.42, respectively. In 2018, 2019 and 2020, Lake Keluke’s phytoplankton diversity was 0.81 ± 0.59, 0.79 ± 0.55 and 0.55 ± 0.61, respectively. Additionally, in 2018, 2019 and 2020, Lake Tuosu’s phytoplankton diversity was 0.83 ± 0.53, 0.48 ± 0.60, and 0.44 ± 0.61, respectively.

### 3.3 RDA analyses in the three lakes

**Figure 3:**
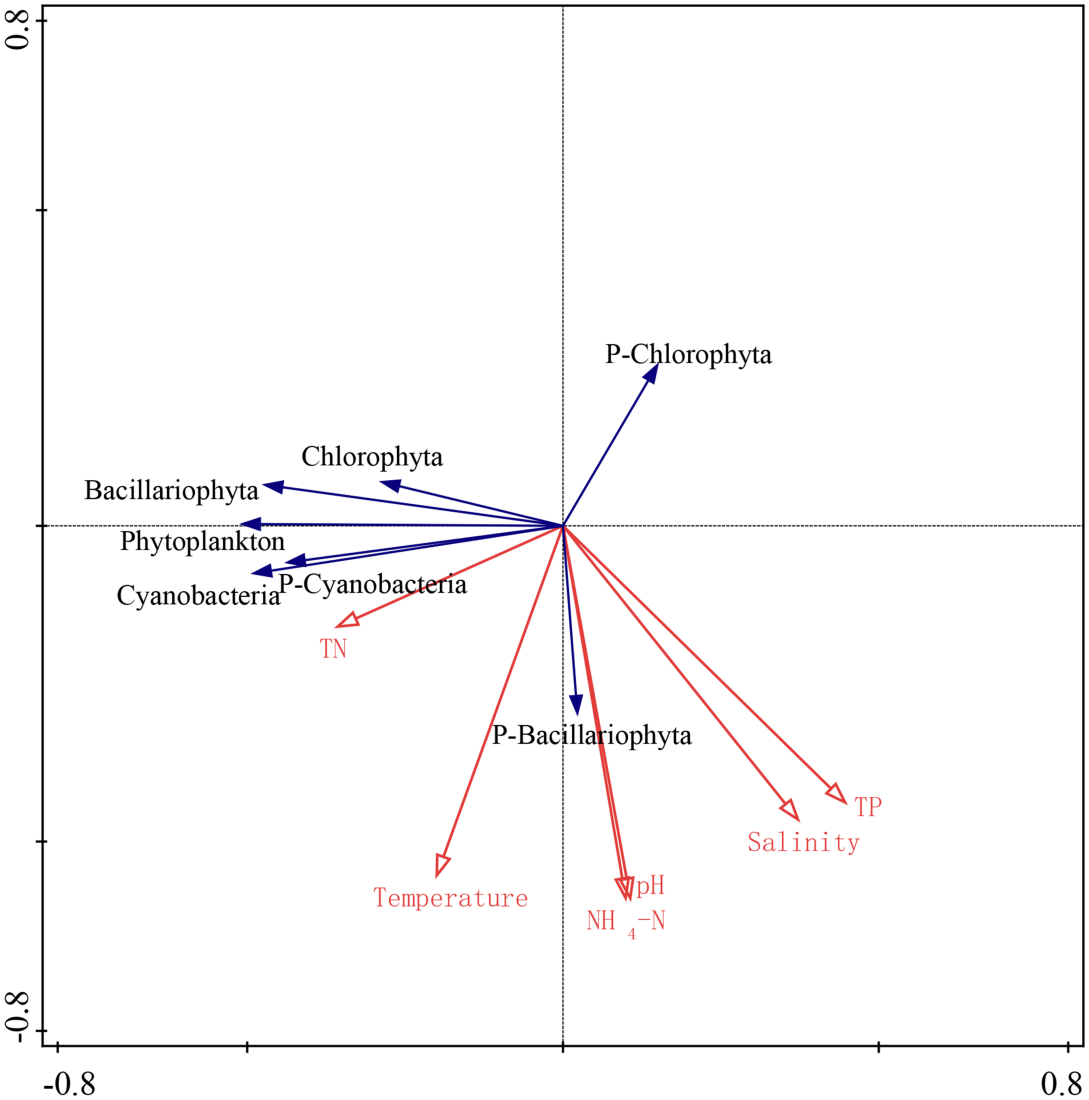
RDA analyses of biological and environmental factors in Lake Qinghai, Lake Keluke and lake Tuosu.

The relationship among phytoplankton and water parameters was analyzed by redundancy analysis (RDA), as shown in Figure 3. Based on the results of the RDA analysis, it can be seen that the eigenvalue of axis 1 is 0.37, and the eigenvalue of axis 2 is 0.19, and the two axes can explain 56.0% of the variation rate. RDA analyses showed that cyanobacteria, Chlorophyta, phytoplankton abundance, and the ratio of cyanobacteria/phytoplankton were positively correlated with temperature in Lake Qinghai, Lake Keluke and Lake Tuosu in 2018–2020 (*p* < 0.05). Bacillariophyta, phytoplankton abundance and the ratio of Chlorophyta/phytoplankton were all negatively correlated with salinity (*p* < 0.05). Meanwhile, cyanobacteria, Bacillariophyta, Chlorophyta and phytoplankton abundance were all negatively correlated with NH_4_-N (*p* < 0.05). Besides, the ratio of Bacillariophyta /phytoplankton and the ratio of cyanobacteria/phytoplankton were all negatively correlated with NH_4_-N (*p* < 0.05).

### 3.4 Linear regression analysis based on annual average values of water parameters and phytoplankton in the three lakes

**Figure 4:**
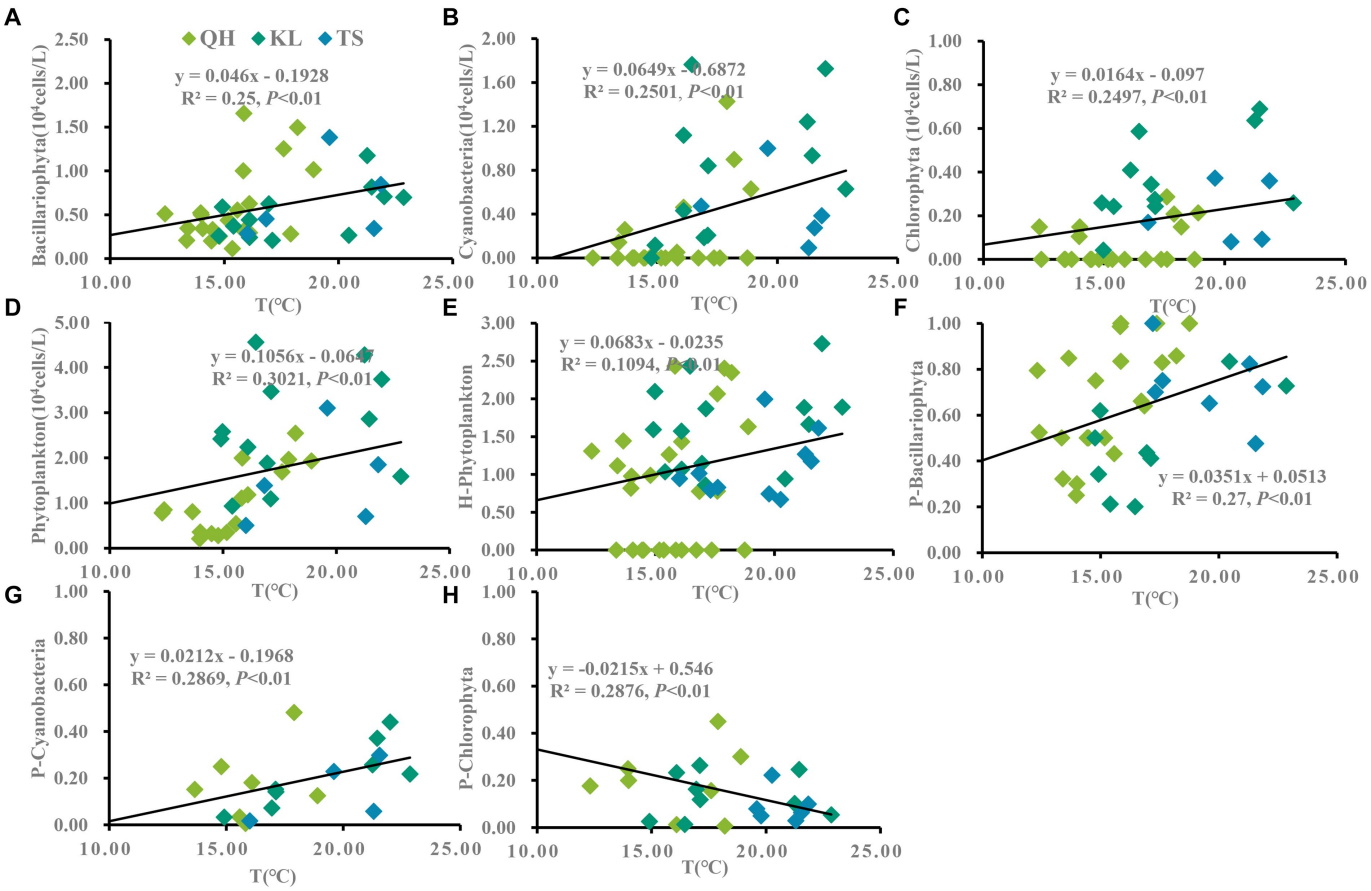
Linear regression models was applied to temperature (T) and phytoplankton based on yearly average data from each site of Lake Qinghai, Lake Keluke and Lake Tuosu. The response of phytoplankton to T were shown in **(A–D)** and response of phytoplankton composition to T were shown in **(E–H)**. Bacillariophyta, cyanobacteria, Chlorophyta and phytoplankton abundance were changed via Log (X + 1). P-i represents the i/phytoplankton ratio based on abundance. H-phytoplankton indicates the biodiversity of phytoplankton.

**Figure 5:**
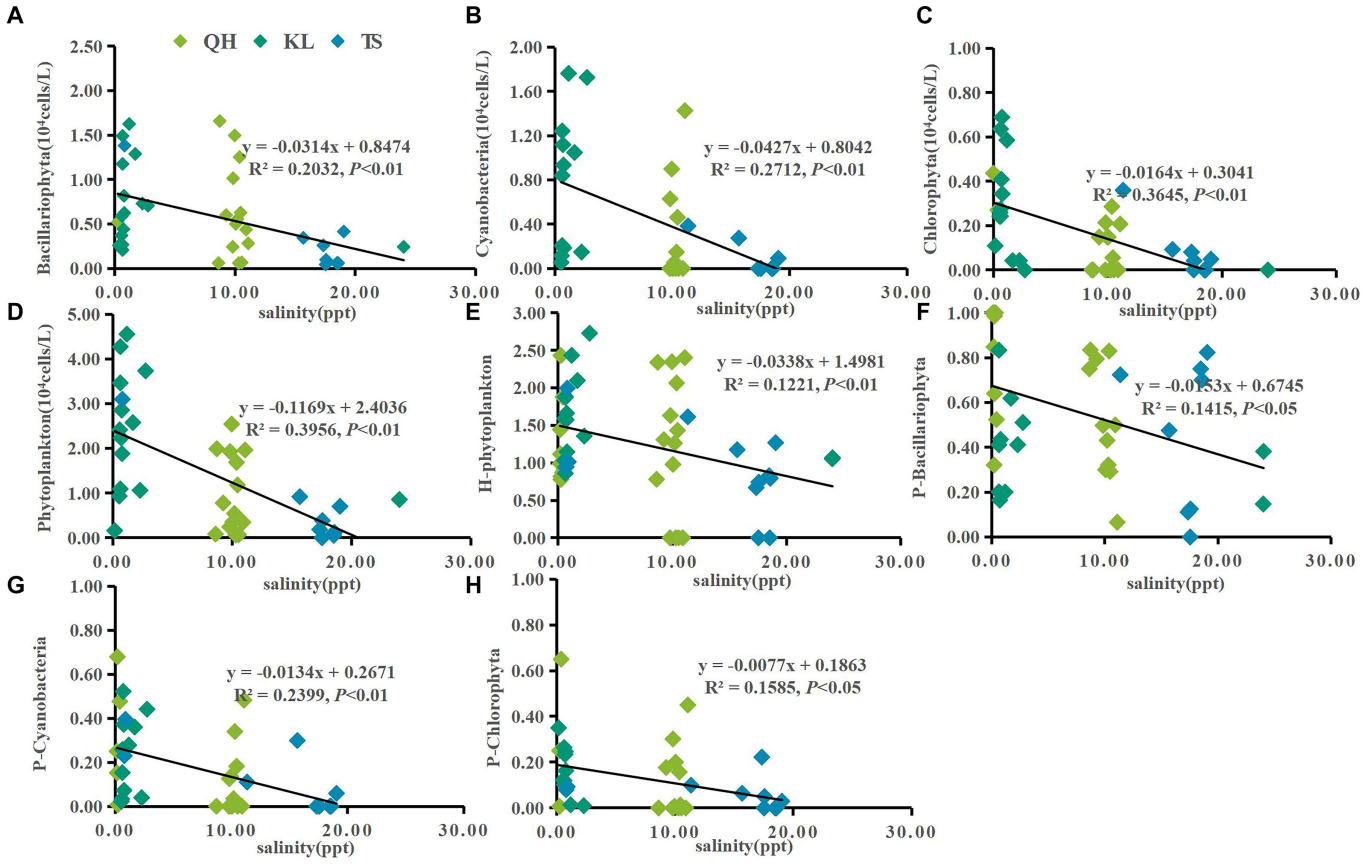
Linear regression models was applied to salinity and phytoplankton based on yearly average data from each site of Lake Qinghai, Lake Keluke and Lake Tuosu. The response of phytoplankton to salinity were shown in **(A–D)** and response of phytoplankton composition to salinity were shown in **(E–H)**. Bacillariophyta, cyanobacteria, Chlorophyta and phytoplankton abundance were changed via the Log (X + 1). P-i represents the i/phytoplankton ratio based on abundance. H-phytoplankton indicates the biodiversity of phytoplankton.

**Figure 6:**
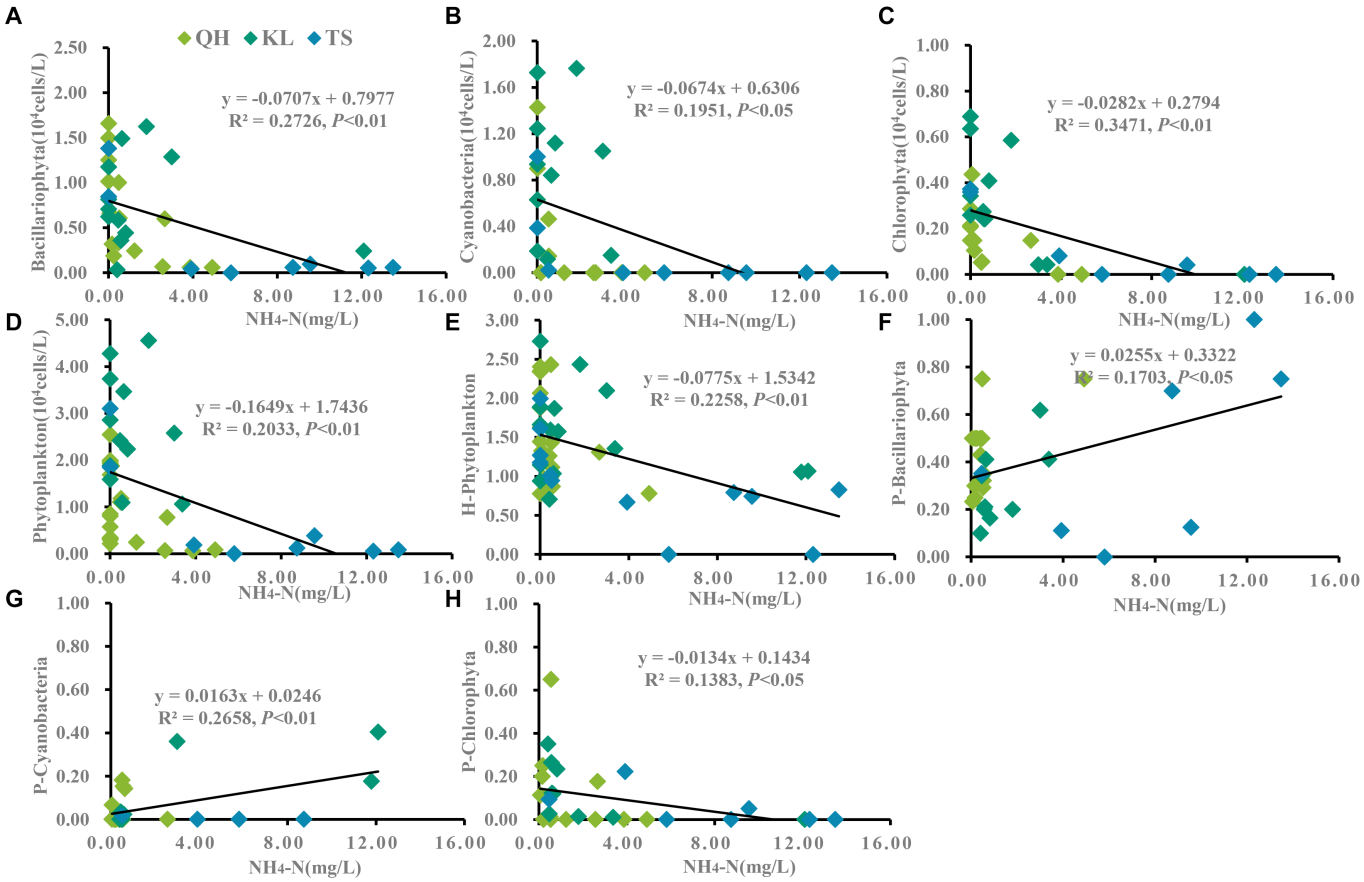
Linear regression models was applied to NH_4_-N and phytoplankton based on yearly average data from each site of Lake Qinghai, Lake Keluke and Lake Tuosu. The response of phytoplankton to NH_4_-N were shown in **(A–D)** and response of phytoplankton composition to NH_4_-N were shown in **(E–H)**. Bacillariophyta, cyanobacteria, Chlorophyta and phytoplankton abundance were changed via the Log (X + 1). P-i represents the i/phytoplankton ratio based on abundance. H-phytoplankton indicates the biodiversity of phytoplankton.

According to the annual average data from Lake Qinghai, Lake Keluke and Lake Tuosu, phytoplankton had clear responses to water parameters, such as temperature, salinity and NH_4_-N in Figures 4–6. Cyanobacteria, Bacillariophyta, Chlorophyta and phytoplankton abundance all increased with temperature with R^2^ = 0.25, 0.25, 0.25 and 0.30, respectively (*p* < 0.01). Additionally, the ratio of cyanobacteria/phytoplankton and the ratio of Bacillariophyta/phytoplankton both increased with temperature (*p* < 0.01).

Significant responses were also observed between phytoplankton and salinity based on the annual average data from Lake Qinghai, Lake Keluke and Lake Tuosu (Figure 5). Cyanobacteria, Bacillariophyta, Chlorophyta and phytoplankton abundance all decreased with salinity under R^2^ = 0.27, 0.20, 0.36 and 0.40, respectively (*p* < 0.01). In addition, the ratio of Bacillariophyta/phytoplankton, the ratio of Chlorophyta/phytoplankton and the ratio of cyanobacteria/phytoplankton all decreased with the salinity (*p* < 0.05). Furthermore, based on the annual average data from the three alpine lakes, phytoplankton had clear response to NH_4_-N (Figure 6). Cyanobacteria, Bacillariophyta, Chlorophyta and phytoplankton abundance declined with NH_4_-N (*p* < 0.05; Figure 6). In addition, the ratio of cyanobacteria/phytoplankton, the ratio of Bacillariophyta/phytoplankton and the ratio of Chlorophyta/phytoplankton all decreased with NH_4_-N (*p* < 0.05).

## 4 Discussion

Previous studies had indicated that the Tibetan Plateau are sensitive to climate change, which was two-three times of the average level (Lan, 2024). In present study, physicochemical parameters variations showed that there were clear responses of aquatic environment to climate change, especially in temperature, salinity and nutrients. This would inevitably affect the phytoplankton genera and community structure, and have a profound impact on the structure and function of the ecosystem in alpine lakes. Previous studies have shown that the increased of temperatures changed the phytoplankton composition and structure (Trombetta et al., 2019), the maximum biomass, and promoted the growth of cyanobacteria and Chlorophyta. Moreover, the temperature elevation can enhance the competitiveness of the dominant genera, while accelerating the process of succession in different trophic phytoplankton in the water, and also making eutrophication water appear the common dominant genera in even heavy eutrophic water (Winder and Sommer, 2012; Yang, 2016). In current study, linear regression analysis showed that cyanobacteria, Bacillariophyta, Chlorophyta and overall phytoplankton all increase with temperature. Besides, the ratio of cyanobacteria/phytoplankton and the ratio of Bacillariophyta/phytoplankton both increased with temperature. Those results indicated that phytoplankton abundance would increase if water temperature rise under climate change. Both cyanobacteria and Bacillariophyta are sensitive to temperature change and the phytoplankton composition would tend to shift toward cyanobacteria and Bacillariophyta in alpine lakes with temperature rise. The inferences were consistent with the results of both temporal and spatial variations in temperature and phytoplankton. The three lakes exhibited cyanobacteria, Bacillariophyta, Chlorophyta and phytoplankton showed significantly higher abundances in 2018 compared to 2019 and 2020. Concurrently, higher average temperatures in 2018 than in the subsequent years. Besides, elevated temperatures particularly benefit cyanobacteria growth. Lake Keluke and Lake Tuosu displayed greater average cyanobacteria abundance and a higher cyanobacteria/phytoplankton ratio compared to Lake Qinghai.

Extreme environmental changes can limit species richness. Halotolerance, which determines species diversity in hypersaline lakes, restricts species diversity. Many saline lakes worldwide exhibit a negative correlation between species richness and salinity. However, hypersaline lakes remain under-studied compared to their freshwater counterparts (Williams et al., 1990; Williams, 1998; Williams, 2002; Larson and Belovsky, 2013). The lakes on Tibetan Plateau may undergo a decline in salinity since the balance of precipitation and evaporation was broken as reflected by the expansion of the surface water in many alpine lakes under climate change (Song et al., 2022). A study by Li et al. (2021) found that salinity was the dominant factor controlling phytoplankton community abundance and biomass in lake systems on the Qinghai**-**Tibet Plateau. Phytoplankton communities were analyzed along salinity, revealing that phytoplankton and overall abundance was significantly and negatively correlated to salinity (*p <* 0.05). High salinity levels make phytoplankton unable to maintain normal cell osmotic pressure, resulting in low phytoplankton abundance and biomass in high-salinity lake. This is consistent with our findings. In this study, cyanobacteria, Bacillariophyta, Chlorophyta and overall phytoplankton abundance tend to decline with increase of salinity. Besides, the ratios of cyanobacteria to phytoplankton, the ratios of Bacillariophyta and Chlorophyta to phytoplankton were decrease with salinity. Those results indicate that the abundance of cyanobacteria, Bacillariophyta and Chlorophyta may increase if the salinity decreased under climate change. Moreover, the low phytoplankton abundance in certain lakes is not solely due to salinity but also nutrients and other growth-limiting elements, such as light and temperature. *Microcystis aeruginosa*, a common freshwater blooming genus, could endure salinities as high as 17.5 for 9 days under laboratory (Preece et al., 2017; Georges des Aulnois et al., 2019). Meanwhile, *Microcystis* blooms toxicity was highest at low salinity in estuary (Lehman et al., 2005). Therefore, the phytoplankton growth could be affected by high salinity, but the salinity was not the determinant factor responsible for the low phytoplankton. In our study, while the salinity in Lake Keluke was below the maximum tolerance observed in laboratory conditions, phytoplankton abundance remained low. Therefore, Lake Keluke does not necessarily have low phytoplankton abundance due to high salinity. Lake Tuosu has a salinity of 13.08 ± 0.4, which may negatively affect various phytoplankton genera. This also verifies that phytoplankton abundance in Lake Tuosu is relatively high in low salinity years.

Despite the salinity change under precipitation augments in alpine lakes, the nutrient had a significant variations due to the increasing amount of nitrogen and phosphorus along with runoff (Wu et al., 2012). Ammonia was one of important compositions in the nutrient, which significantly affect lake phytoplankton biomass (Dai et al., 2008; Drath et al., 2008). Its concentration typically depends on environmental pH, salinity and temperature. At a pH below 8.75, ammonia predominantly exists in the form of NH_4_-N. There is a significant relationship between ammonia concentration and organisms, directly affecting their normal activities (Zhao et al., 2020). In lake ecosystem, ammonia serves as a nutrient salt and is a key indicator for monitoring environmental pollution, reflecting the survival of aquaculture animals and phytoplankton (Wu et al., 2006; O’Connor Šraj et al., 2018; Zhao et al., 2020). Previous studies have indicated that phosphorus substantially enhances total phytoplankton biomass and growth rates, with nitrogen exerting secondary effects. However, excessive nitrogen can be detrimental to phytoplankton (Dai et al., 2008; Drath et al., 2008). It has been noted that cyanobacteria and Chlorophyta typically have greater NH_4_-N tolerance thresholds than Bacillariophyta. Therefore, cyanobacteria and Chlorophyta are less affected by high concentrations of NH_4_-N and are not outcompeted by other taxa when NH_4_-N is the sole nitrogen source (Esparza et al., 2014). In this study, correlation analyses showed that NH_4_-N with cyanobacteria, Bacillariophyta, Chlorophyta and overall phytoplankton abundance were all significantly negatively correlated. This was also found in Lake Keluke before that the both TN and NH_4_-N had very high concentration and were negative for phytoplankton growth (Wang et al., 2018). NH_4_-N is the most readily absorbed nitrogen source by phytoplankton since the absorption of NH_4_-N by phytoplankton is passive diffusion and does not require energy consumption. However, the absorption of other forms of nitrogen is active transport and requires energy consumption. Previous articles have indicated that as NH_4_-N decreases, inflow to lake NH_4_-N concentrations also decreases, thereby reducing or eliminating phytoplankton sensitivity to NH_4_-N. For current highland lakes such as Keluke and Tuosu, ammonia nitrogen levels are high and cyanobacteria levels are gradually rising, threatening the survival of fish (Dugdale et al., 2013). In aquaculture, ammonia produced by fish is absorbed by phytoplankton. With the proliferation of phytoplankton, ammonia levels increase continuously, leading to water body eutrophication and moderate fish kills (Collos and Harrison, 2014). Meanwhile, ammonia is a major water pollutant and its rapidly increase escalates the mortality rate of fish and shrimp, causing substantial economic losses to farmers (Zhao et al., 2020).

Algal bloom in plateau lakes are increasingly frequent globally, especially in nutrient-poor lakes (Zhang et al., 2020). Alterations in nutrients such as nitrogen and phosphorus can affect phytoplankton growth. Several studies have shown that the addition of phosphorus significantly increases phytoplankton biomass and growth rates (Carpenter et al., 1998b). TN is a crucial indicator for assessing a lake’s eutrophication level and is vital for phytoplankton growth and metabolism. Inappropriate TN content can affect phytoplankton growth and community structure succession (Paparazzo et al., 2017; Le et al., 2019). In this study, high TN concentrations were observed in all three lakes, with little variation in TP concentrations. The dominant phytoplankton’s growth was not nitrogen-limited, but phosphorus limited, with TP levels considerably below 0.06 mg/L. Hence, TP did not determine the differences in phytoplankton abundance across the lakes. In Lake Taihu, where ammonia levels peak during spring and winter (non-growing season), bloom-forming *Microcystis* spp. might experience growth inhibition due to NH_4_-N toxicity (Dai et al., 2012). In contrast, Chlorophyta, less sensitive to ammonia, could flourish (Xu et al., 2010). Excessive nitrogen can be toxic to phytoplankton (Dai et al., 2008). In all three alpine lakes of this study, phytoplankton in both Lakes Keluke and Tuosu struggled to thrive under elevated NH_4_-N conditions. Thus, NH_4_-N is key to regulating the distribution of cyanobacterial blooms and common algae group, both as a nutrient and a toxin in cyanobacterial blooms (Dai et al., 2012).

The sensitivity of phytoplankton to the aquatic environment leads to the vulnerability of their community characteristics to changes in aquatic systems. The dominant phytoplankton groups were cyanobacteria, Bacillariophyta and Chlorophyta in the three lakes during the survey. The moderate temperature and nutrient levels of Lake Qinghai make it easier to form dominant taxa Bacillariophyta. In general, *Synedra* sp. and *Navicula* sp. (Bacillariophyta) were the dominant genera in Lake Qinghai from 2018 to 2020, whereas the dominant genus *Spirogyra* sp. of the Chlorophyta appeared in the summer of 2019 up to 15% of the total phytoplankton. This was due to the high NH_4_-N content of Lake Qinghai in the summer of 2019, since Chlorophyta are more likely to grow in a nitrogen-rich, phosphorus-rich, and temperature-suitable condition (Gamier et al., 1995). This phenomenon could be seen in Lake Tuosu in the spring of 2020. The seasonal phytoplankton composition of Lake Keluke showed a clear seasonal succession, with moderate spring temperatures in 2018 and 2019, resulting in the emergence of Bacillariophyta as dominant phytoplankton group, then the dominant was evolved from Bacillariophyta to cyanobacteria during the summer of 2018–2019 and the spring of 2020 due to increasing temperatures, include the genera *Dolichospermum* sp., *Oscillatoria* sp. and *Pseudoanabaena* sp., which preferred high temperatures (Nalley et al., 2018). Cyanobacteria reproduce fast in the high temperature season, while Bacillariophyta have strong adaptability in the low temperature season. This is in accordance with the change process of Bacillariophyta in spring and peak cyanobacteria in summer in Lake Keluke (Chomérat et al., 2007; Ding et al., 2023). In the present study, phytoplankton were dominated by cyanobacteria in Lake Keluke in the summer of 2019 and spring of 2020, and in Lake Tuosu in the summer of 2019. This was consistent with the previous research that cyanobacteria tended to dominate when the water body was high in nutrients (Sun, 2023).

The response of phytoplankton in alpine lakes to environmental factors is mainly expressed in changes of temperature increase, salinity, and nutrients. The impact of rising temperature is notably significant in the lakes of the Tibetan Plateau, and precipitation tends to increase in the east and north. As a result, the lakes in the Tibetan Plateau experience a warm and wet climate (Zong-Xue et al., 2006; Yao et al., 2007; Liao et al., 2013; Lin et al., 2017). This leads to increases in lake water temperature and nutrients, but a decrease in salinity, potentially facilitating the growth of nuisance algae such as cyanobacteria (Jeppesen et al., 2010). Lake Qinghai had experienced a gradual increase of phytoplankton abundance in the past decades. The phytoplankton average abundance was 7.0 × 10^4^ cells/L in the summer of 1961–1962 (Yao et al., 2007). The abundance was up to 1.22 × 10^5^ cells/L during 2006–2010 (Yao et al., 2011). In present study, the phytoplankton abundances was upward 2.9 × 10^5^ cells/L in 2020, which more than two times higher than 2006–2010. Both the ratios of Bacillariophyta and cyanobacteria accounted for phytoplankton were increased from 2006–2010 to 2018–2010. The ratios of Bacillariophyta reached 77% from 71% and the ratios of cyanobacteria was up to 14% from 6% (Yao et al., 2011). In Lake Keluke, there was an increase of 22 phytoplankton species in 2018–2020 compared to 2007 (Wang and Li, 2014), and cyanobacteria are the majority. The phytoplankton average densities were 3.2 × 10^5^ cells/L in 1977–1980 (Zhao et al., 1990), which were similar with 2018–2020. However, the ratios of cyanobacteria were up to 46% (2018–2020) from 10% (2007).

Lake Qinghai, renowned as a popular tourist destination, attracts a significant number of residents and tourists. Lake Keluke, additionally, stands as a small salty water in Qinghai Province. The density of residents, coupled with overgrazing and surface farming, is progressively damaging the aquatic vegetation of the wetlands and thereby continuously promoting eutrophication in the lake. A study focused on a typical brackish lake with ample nutrients on the Qinghai-Tibet Plateau found that with increased eutrophication due to climate change, phytoplankton blooms might also emerge (Wu et al., 2021). Human activity on the plateau has steadily increased with ongoing socio-economic growth, impacting the water quality and evolution of many plateau lakes. The Qinghai-Tibet Plateau is a habitat for numerous rare birds and native plateau fish. Thus, managing brackish lakes on the Qinghai-Tibet Plateau should be a priority. Furthermore, the primary sources of nitrate in the groundwater near lakes such as Lake Qinghai and Lake Tuosu originate from animal feces and sewage (Li et al., 2021). Therefore, establishing buffer zones is crucial for the effective planning, design, and management of livestock waste and wastewater from the grazing industry. The ongoing expansion of tourism at Lake Qinghai and aquaculture at Lake Keluke, while generating increased economic revenue, also undeniably exacerbates the ecological burden on these scenic areas. This added pressure impairs the self-regulation capabilities of their ecological environments. Consequently, it is imperative for relevant management authorities to undertake effective planning, commit to the protection of the ecological environment, and embrace a strategy of sustainable development.

## 5 Conclusion

There were significant changes in physicochemical parameters and phytoplankton in the three lakes. The significant variations of parameters were mainly in temperature, salinity and nutrients. Phytoplankton presented different successions in the three lakes. Bacillariophyta was predominant in Lake Qinghai from 2018 to 2020, with *Synedra* sp., *Navicula* sp., *Cymbella* sp. and *Achnanthidium* sp. emerging altertively as the dominant genera. Lake Keluke alternated between being dominated by cyanobacteria and Bacillariophyta during the same period. The genus *Dolichospermum* sp., a cyanobacteria, was dominanted in the summer of 2018 and 2019 and in the spring of 2020. In Lake Tuosu, Bacillariophyta was predominant from 2018 to 2020, except in the summer of 2019, which was dominated by cyanobacteria. The dominant genera *Synedra* sp., *Oscillatoria* sp., *Pseudoanabaena* sp., *Chromulina* sp. and *Achnanthidium* sp. were appeared successively. Both temporal and spatial variations showed that phytoplankton had a relative high abundance under high temperature, especially cyanobacteria. Linear regression analysis showed the abundance of cyanobacteria, Bacillariophyta, Chlorophyta and overall phytoplankton increased with temperature and decreased with salinity and NH_4_-N. Besides, the ratios of cyanobacteria, and the ratios of Bacillariophyta accounted in total phytoplankton increased with temperature. These findings suggest that cyanobacteria and phytoplankton abundance, especially Bacillariophyta may have an increase tendency in the three alpine lakes under warm and wet climate.

## Data availability statement

The original contributions presented in the study are included in the article/supplementary material, further inquiries can be directed to the corresponding author.

## Author contributions

PG: Data curation, Formal analysis, Writing – original draft. JJ: Funding acquisition, Investigation, Writing – review & editing. DQ: Writing – review & editing. QG: Writing – review & editing. CZ: Writing – review & editing. XY: Writing – review & editing. MN: Writing – review & editing. DL: Writing – review & editing. YL: Writing – review & editing.
